# Abnormal dynamic functional connectivity in Alzheimer’s disease

**DOI:** 10.1111/cns.13387

**Published:** 2020-05-06

**Authors:** Yue Gu, Ying Lin, Liangliang Huang, Junji Ma, Jinbo Zhang, Yu Xiao, Zhengjia Dai, Alzheimer's Disease Neuroimaging Initiative

**Affiliations:** ^1^ Department of Psychology Sun Yat‐sen University Guangzhou China

**Keywords:** Alzheimer's disease, dynamic, state, temporal variability

## Abstract

**Aims:**

Alzheimer's disease (AD) is a progressive neurodegenerative disorder. Previous studies have demonstrated abnormalities in functional connectivity (FC) of AD under the assumption that FC is stationary during scanning. However, studies on the FC dynamics of AD, which may provide more insightful perspectives in understanding the neural mechanisms of AD, remain largely unknown.

**Methods:**

Combining the sliding‐window approach and the *k*‐means algorithm, we identified three reoccurring dynamic FC states from resting‐state fMRI data of 26 AD and 26 healthy controls. The between‐group differences both in FC states and in regional temporal variability were calculated, followed by a correlation analysis of these differences with cognitive performances of AD patients.

**Results:**

We identified three reoccurring FC states and found abnormal FC mainly in the frontal and temporal cortices. The temporal properties of FC states were changed in AD as characterized by decreased dwell time in State I and increased dwell time in State II. Besides, we found decreased regional temporal variability mainly in the somatomotor, temporal and parietal regions. Disrupted dynamic FC was significantly correlated with cognitive performances of AD patients.

**Conclusion:**

Our findings suggest abnormal dynamic FC in AD patients, which provides novel insights for understanding the pathophysiological mechanisms of AD.

## 1 INTRODUCTION

Alzheimer's disease (AD) is a progressive neurodegenerative disease that reduces the quality of patients’ daily life as their cognitive and functional abilities decline.^1^ A growing number of studies suggest that the disruption of functional connectivity (FC) among brain regions may be an early outcome of neurotoxic β‐amyloid (Aβ) aggregation in AD.^2^ Abnormal FC may eventually lead to a decline in high‐level cognitive functions.^3^

Recent advances in neuroimaging techniques provide the opportunity to study disconnections (ie, a disruption of FC) in AD in vivo. Studies using resting‐state functional magnetic resonance imaging (R‐fMRI) have shown that statistical correlations of spontaneous activities exist between functionally correlated brain regions.^4^ In relation to AD, abnormal FC has been found in functional hub regions,^5^, ^6^ neural circuits,^7^, ^8^ and the whole‐brain level.^9^, ^10^ Despite these advances, previous studies mostly assumed that the FC was constant during MRI scanning, ignoring its dynamic nature.^11^, ^12^

Compared to stationary FC, dynamic FC allows investigating the R‐fMRI time series on a much finer scale (eg, at specific time points or within predefined time windows), which provides two exclusive advantages. On one hand, dynamic FC facilitates the observation of details that are averaged out in stationary FC and may offer greater insight into the fundamental mechanisms of FC. On the other hand, dynamic FC enables the capture of spontaneously reoccurring FC patterns (ie, FC states), which is essential for understanding the temporal variability in the intrinsic organization of the brain. Based on these advantages, researchers have found that dynamic FC is a potential sensitive biomarker for neuropsychiatric disorders, such as schizophrenia,^13^ autism,^14^ and Parkinson's disease.^15^ To our knowledge, only a few studies have examined the FC dynamics associated with AD.^16^, ^17^, ^18^ Among these studies, Jones et al^17^ focused on demonstrating the nonstationary nature of the brain's modular organization and only used AD for validation; Fu et al^16^ and Schumacher et al^18^ were more concerned about comparing the FC dynamics profile (ie, dwell time in each state) of AD with that of the other subtypes of dementia. Besides, all these studies were conducted based on functional networks constructed from independent component analysis, which restricts the resolution of their findings. Hence, comprehensive and in‐depth investigations of the unique features and temporal properties of FC dynamics in AD compared with healthy controls (HCs) are still lacking.

To fill this gap, we first employed the sliding‐window approach and the *k*‐means algorithm to identify FC states that reoccur over time. Then, the FC patterns under each state and the temporal properties of the FC states were explored. Furthermore, we evaluated the temporal variability of regional FC along the entire time series. Finally, the correlation analyses between the above indicators of FC dynamics and AD cognitive performances were performed.

## 2 MATERIALS AND METHODS

### 2.1 Participants

Twenty‐six AD patients and twenty‐six age‐ and sex‐matched HCs with resting‐state fMRI data from the Alzheimer's Disease Neuroimaging Initiative (ADNI)‐2 database (http://adni.loni.usc.edu/) were used in this study. The AD patients met the criteria of the National Institute of Neurological and Communicative Disorders and Stroke and the AD and Related Disorders Association (NINCDS/ADRDA) for probable AD. Disease severity was assessed in AD using the Clinical Dementia Rating (CDR). All participants underwent an examination of Mini‐Mental State Examination (MMSE) and Neuropsychiatrie Inventory (NPI). The inclusion criteria for HC were as follows: (a) no neurological or psychiatric disorders, such as mild cognitive impairment, depression, or epilepsy; (b) no abnormal findings, such as infarction or focal lesion in conventional brain MR imaging; and (c) MMSE score beyond 27. The demographic and clinical data of the participants are listed in Table 1.

**Table 1:** Participant demographic and clinical characteristics

	Healthy controls	Patients with Alzheimer's disease	*P*‐value
Gender	11 M, 15 F	12 M, 14 F	.780
Age (SD)	75.7 (6.2)	74.6 (6.5)	.832
MMSE (SD)*	29.04 (1.33)	21.18 (3.2)	<10^‐3^
CDR*	0 (n = 19), 0.5 (n = 2), 1 (n = 1)	0.5 (n = 6), 1 (n = 15), 2 (n = 1)	<10^‐3^
NPI (SD)*	0.71 (1.31)	4.59 (3.92)	<10^‐3^

Note

Four AD patients had no MMSE, CDR, or NPI score. Four HC had no CDR score, while three of whom neither had MMSE score. Excepting these four HC, five other HCs had no NPI score. The two‐sample two‐tailed t‐test was performed to examine between‐group differences in age and MMSE, Mann‐Whitney U‐test was used for NPI, and chi‐square test was performed for gender and CDR.

The asterisk indicates a significant between‐group difference (*P* < .05).

Abbreviation: AD, Alzheimer's disease; CDR, Clinical Dementia Rating; HC, healthy control; MMSE, Mini‐Mental State Examination; NPI, Neuropsychiatrie Inventory.

### 2.2 Data acquisition and preprocessing

All participants were scanned on a 3.0 T Philips scanner. MRI acquisitions were performed according to the ADNI acquisition protocol.^19^ R‐fMRI was obtained using an echo‐planar imaging (EPI) sequence and the following parameters: repetition time (TR) = 3000 ms, echo time (TE) = 30 ms, flip angle = 80°, number of slices = 48, slice thickness = 3.313 mm, voxel size = 3 mm × 3 mm × 3 mm, voxel matrix = 64 × 64, and total volume = 140. Image preprocessing was carried out using the Data Processing Assistant for Resting‐State fMRI (DPARSF) toolbox and SPM8^20^ (http://www.fil.ion.ucl.ac.uk/spm). For each participant, the preprocessing steps included discarding the first five volumes, correcting head motion, normalizing to the Montreal Neurological Institute (MNI) template, resampling to 3 × 3 × 3 mm^3^, regressing out the nuisance variables (Friston's 24 head motion parameters, global signal, white matter, and cerebrospinal fluid signals), and filtering (0.01 Hz–0.08 Hz).

### 2.3 Construction of dynamic functional networks

Dynamic functional brain network construction was carried out using graph theoretical network analysis^21^ (GRETNA, http://www.nitrc.org/projects/gretna/). The nodes were defined by 625 similar‐size brain regions according to the automated anatomical labeling (AAL) atlas landmark.^22^, ^23^ Dynamic FC was estimated with the widely used sliding‐window method.^12^, ^15^ Specifically, we computed Pearson correlation coefficients between each pair of nodes using the time course segment within a time window. The window length was set as 10 TRs (ie, 30 s) according to previous studies.^24^, ^25^ By moving the time window forward by a step of one TR (ie, 3 s), we obtained a total of 126 625 by 625 symmetric correlation matrices for each participant. With each correlation matrix representing the FC pattern in one time window, these correlation matrices could capture the dynamic changes of FC during the resting‐state scan period and were used as inputs for further analyses of FC states and temporal variability.

### 2.4 Detection of dynamic functional connectivity states

To assess the architecture and the frequency of reoccurring FC patterns, the *k*‐means algorithm^26^ was applied to group correlation matrices according to their L1 distances. First, a subsampling procedure was carried out to facilitate determining the optimal cluster number and the initial cluster centroids. Similar to EEG microstate analysis,^26^ the subsampling procedure first chose the correlation matrices with locally maximal FC variance (ie, matrices whose L1‐norm was 1.5 standard deviation away from the L1‐norm of the mean correlation matrix) for each participant.^12^ Thus, 767 matrices of the 52 participants (14.3 ± 3.3 matrices per AD patient, 15.2 ± 3.5 matrices per HC) were obtained. Second, the *k*‐means algorithm was used to group these 767 matrices into *k* clusters. To determine the optimal cluster number, we varied *k* from two to nine and repeated the *k*‐means procedure 100 times for each *k* value. The validity of the clustering results was evaluated using the Silhouette score and the Calinski‐Harabasz index.^27^ The elbow criterion based on the Silhouette score and the peak value of the Calinski‐Harabasz index both indicated three as the most appropriate cluster number (*k* = 3). Finally, initiated by the three cluster centroids obtained from the 767 matrices, the *k*‐means algorithm was further used to group the 6552 correlation matrices derived from all participants into three clusters (ie, dynamic FC states).

Based on the dynamic FC states identified from *k*‐means clustering, we calculated a participant‐specific FC matrix for each participant at each state. Specifically, each element in the matrix was the median of the corresponding elements in the participant's matrices belonging to one state. Then, the differences between AD and HC in the participant‐specific FC matrices were evaluated for each state. We also examined the between‐group differences in the temporal properties of the dynamic FC states from three aspects, including the mean dwell time of each state (ie, the average number of windows that participants spent on one state), the mean number of state transitions (ie, the average number of transitions that occurred between any two states), and the distribution of transition frequency (ie, the fraction of state transitions with a specific source and target).^15^

### 2.5 Temporal variability of regional functional architecture

After investigating the differences in FC dynamics between the AD and HC groups from the perspective of dynamic FC states at the whole‐brain level, we further investigated the between‐group differences from the perspective of the temporal variability of FC in each brain region. Specifically, for each participant, we first measured the temporal stability of FC in brain region *k* by the average Pearson correlation coefficient between the *k*‐th row of every two correlation matrices. Then, the temporal variability *V_k_* of region *k* can be described by one minus the temporal stability,^28^ that is,

$$V_{k}=1-\frac{\sum_{i\neq j}\rho_{F_{i,k},F_{j,k}}}{n\times \left(n-1\right)},$$

where n = 126 is the total number of windows and $\rho_{F_{i,k},F_{j,k}}$ is the Pearson correlation coefficient between the FC profiles of region *k* in the correlation matrices derived from the *i*‐th and the *j*‐th windows (*i*, *j* = 1, 2, …, *n*; *i* ≠ *j*; *k* = 1, 2, …, 625).

### 2.6 Statistical analyses

When performing data statistics, we firstly adopted the Lilliefors test to test the distribution of continuous variables for normality. The two‐sample t‐test or general linear model (GLM) analysis was used accordingly for normally distributed data, whilst the Mann‐Whitney U‐test was used for non‐normally distributed data. More specifically, to compare the demographic and clinical data of the participants between AD and HC, the two‐sample t‐test, chi‐square test, and Mann‐Whitney *U*‐test were used as appropriate. To evaluate the difference in the participant‐specific FC matrix between AD and HC, the GLM analysis was performed in a univariate manner with age and gender as covariates [*P* < .05, false discovery rate (FDR) corrected]. After identifying significantly increased/decreased FC in AD compared to HC, the receiver operating characteristic (ROC) analysis was performed to confirm their effectiveness for distinguishing AD from HC. For calculating the temporal properties’ differences between AD and HC, the Mann‐Whitney U‐test was used after controlling age and sex (*P* < .05, FDR corrected). The between‐group comparison of nodal temporal variability (ie, *V_k_*) was performed using the GLM with age and sex controlled (*P* < .05, FDR corrected). Finally, to investigate whether FC dynamics were related to the clinical performance of AD, we set age and sex as controlled variables and computed the partial correlation coefficients between the clinical measures (ie, MMSE and NPI) and the following three indicators: (a) the median strength of FC with significant between‐group differences at each state; (b) the three temporal properties of the dynamic FC states (ie, mean dwell time, mean transition number, and transition frequencies between states); and (c) the temporal variability of the regions that exhibited significant between‐group differences.

### 2.7 Validation analysis: Effect of head motion

Recent studies have suggested that head motion can produce a marked influence on R‐fMRI.^29^, ^30^ To validate our results, we added bad time points as an additional regressor into the nuisance covariate regression model, with the threshold of bad time points set as the framewise displacement of head motion above 0.5 mm as well as one back and two forward neighbors.^31^ Then, we reanalyzed our data to examine our main results.

## 3 RESULTS

### 3.1 Identification of dynamic functional connectivity states

**Figure 1:**
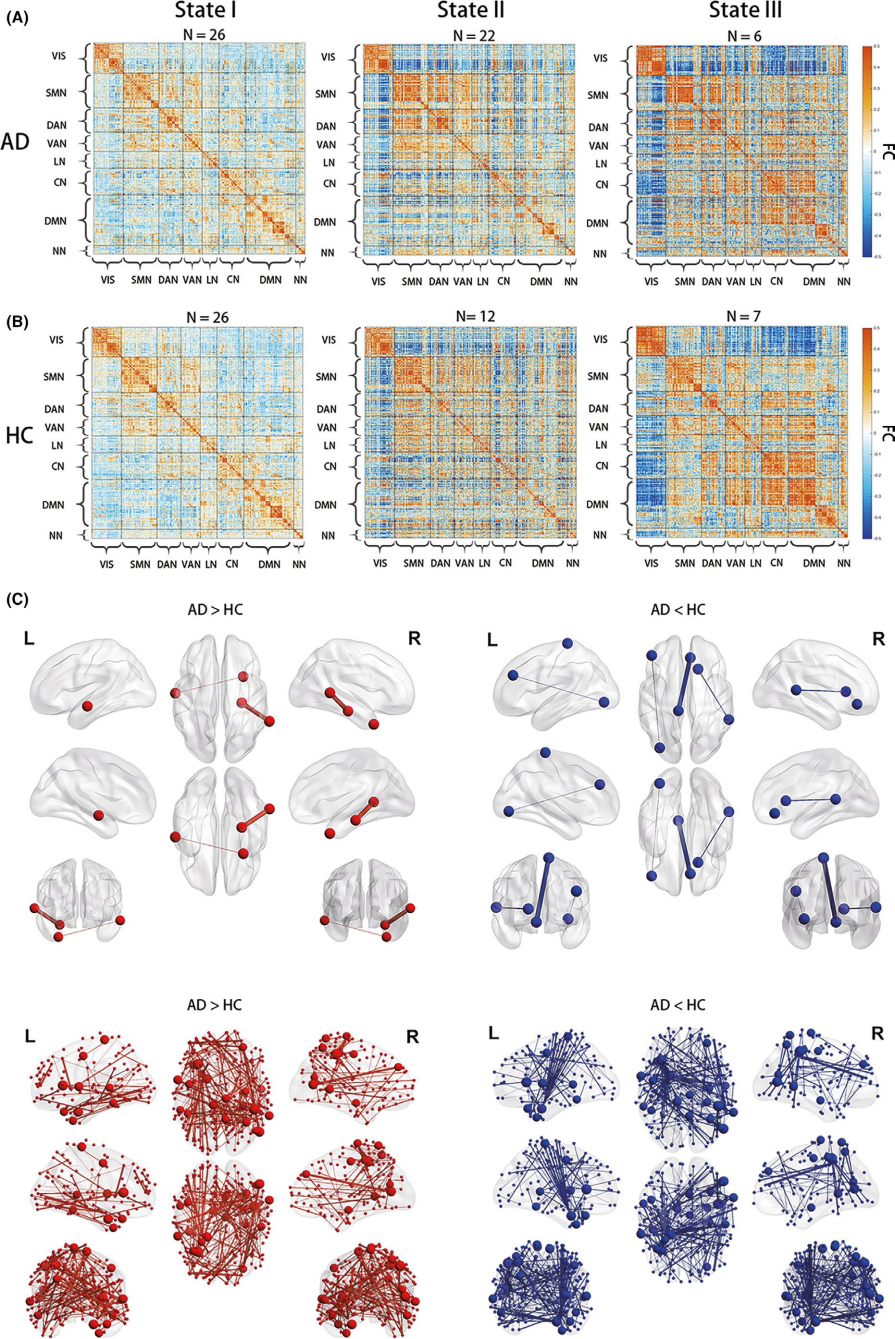
Dynamic functional connectivity states and their between‐group comparisons. (A) The median FC matrix of each state in each group. N is the number of participants that the corresponding state occurred in. Each matrix is organized according to the functional modules extracted by Yeo et al,^54^ which divides the 625 regions into seven networks and one uncertain part. (B) Between‐group difference of functional connectivity in State I (two‐sample t‐test, *P* < .05, FDR corrected). (C) Between‐group difference of functional connectivity in State II (two‐sample t‐test, *P* < .05, FDR corrected). For both of (B) and (C), the significantly increased FC in AD compared to HC (ie, 'AD > HC') was denoted in red, while the significantly decreased FC (ie, 'AD < HC' was denoted in blue. The line width represents the t‐value of between‐group comparison, and the node size represents the number of edges of significant between‐group difference. VIS = visual network; SMN = somatomotor network; DAN = dorsal attention network; VAN = ventral attention network; LN = limbic network; CN = control network; DMN = default mode network; and NN = uncertain network

Using the *k*‐means algorithm, we identified three reoccurring FC states and found that windows were more likely to be in States I and II but less likely to be in State III (Figure 1A). Significant between‐group differences were found in the FC of States I and II (*P* < .05, FDR corrected, Figure 1B,C). In State I, five edges exhibited significant between‐group differences (Figure 1B). Among them, the two significantly increased edges ('AD > HC') connected the right parahippocampal gyrus with the right medial temporal cortex and left medial temporal cortex with the right medial temporal pole. The other three significantly decreased edges ('AD < HC') were between the left paracentral lobe and the right medial temporal cortex, between the left paracentral lobe and the right medial orbitofrontal cortex, and between the left medial frontal cortex and the left inferior occipital cortex. In State II, a total of 436 edges were found to have significant between‐group differences, most of which were associated with the frontal cortex (eg, superior frontal cortex and middle frontal cortex), temporal cortex (eg, middle temporal cortex and hippocampus), insula, and amygdala (Figure 1C).

**Figure 2:**
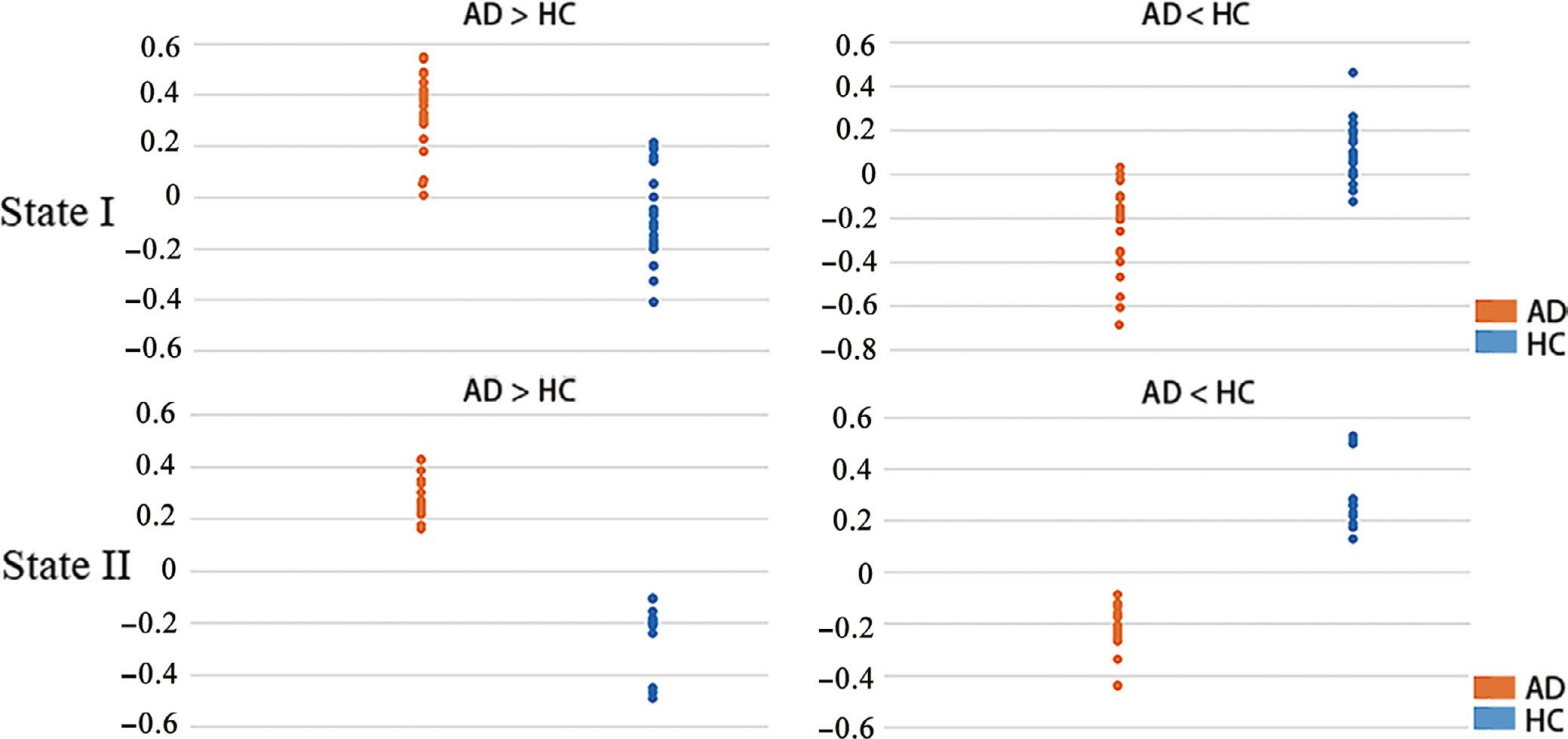
Comparison of the FC with significant between‐group differences. 'AD > HC' indicates the category of FC significantly increased in AD compared to HCs, while 'AD < HC' indicates the opposite category. Each dot in the graphs represents the median strength of the corresponding FC category across all the related correlation matrices in one participant

Intriguingly, detailed examinations of the FC with significant between‐group differences in States I and II found that their signs were consistently opposite between AD and HC (Figure 2). This finding suggested that FC with significant between‐group differences may be useful for distinguishing AD and HC. As a validation, we performed ROC analyses that classified AD and HC according to the median strength of the significantly different (either increased/decreased) FC in each state as input features. The area under the ROC curve (AUC) was above 0.95, suggesting that abnormalities in dynamic FC could serve as potential biomarkers for distinguishing AD from HC.

### 3.2 Analyses of the temporal properties of dynamic functional connectivity states

**Figure 3:**
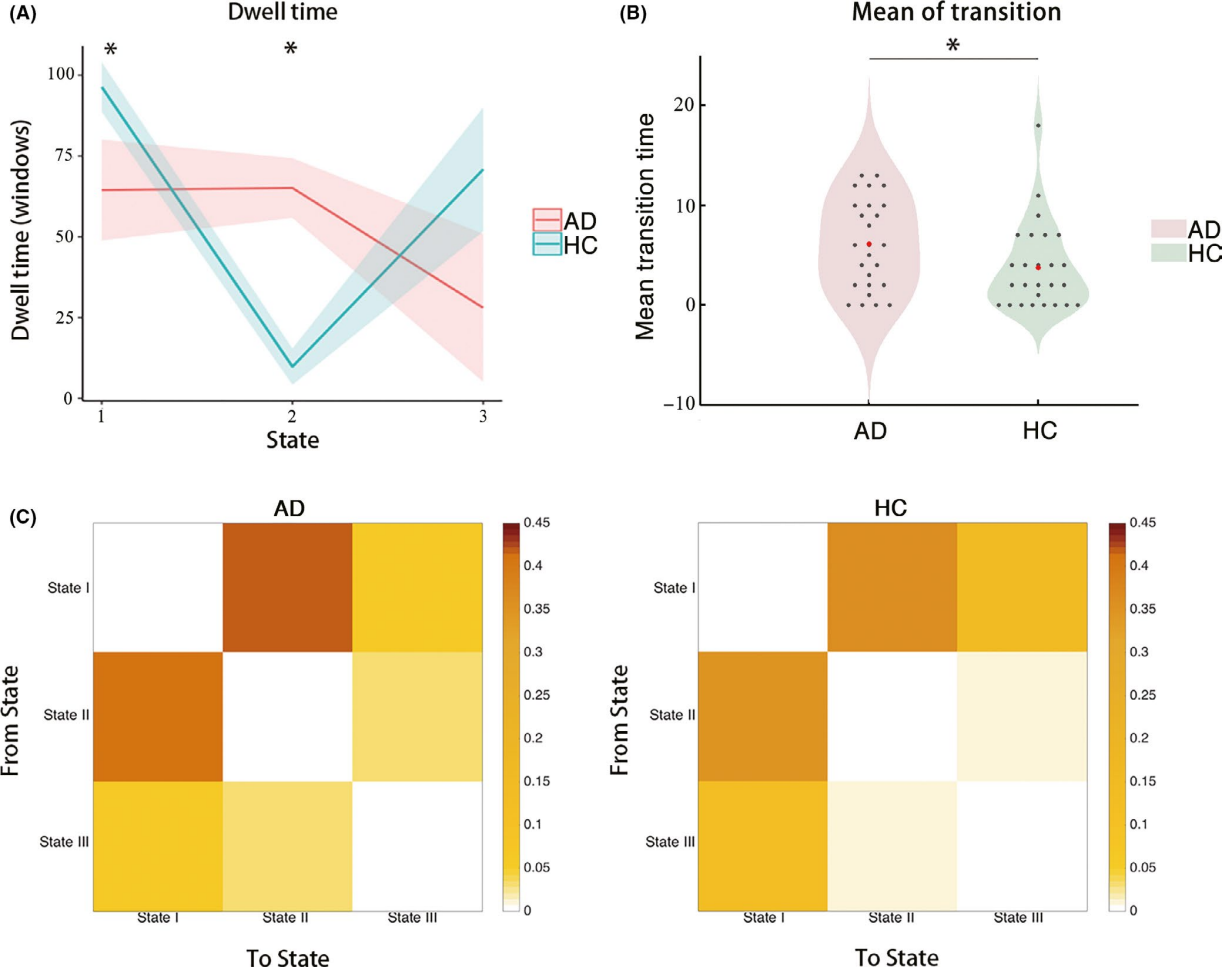
Comparison of the temporal properties of the dynamic FC states between the AD and HC groups. (A) Comparison of the mean dwell time of each state, where the shadow indicates the standard error of mean (SEM) over the corresponding group and the asterisk indicates significant between‐group difference (*P* < .05, FDR corrected). (B) Comparison of the mean transition number, where each black dot indicates the mean transition number of each participant, the red dot indicates the mean transition time of the corresponding group, and the asterisk indicates that significant difference was found between the AD and HC groups (*P* < .05, FDR corrected). (C) Mean transition frequencies within AD and HC group

Figure 3A shows that significant between‐group differences were found in the mean dwell time of States I and II but not State III. Specifically, among the three states, AD had significantly longer mean dwell time in State II than HC (AD: 65.1 ± 9.8; HC: 22.9 ± 5.6, *P* = .015). In contrast, AD had significantly shorter mean dwell time in State I than HC (AD: 64.5 ± 9.2; HC: 96.3 ± 7.7, *P* = .011). Besides, by visual inspection, the within‐group comparison of the mean dwell time in the three states showed that State I and State II were the main states of HC and AD, respectively. Figure 3B shows that AD had significantly more state transitions than HC (AD: 6.1 ± 0.89; HC: 3.7 ± 0.84, *P* = .010). The distribution of state transition frequencies is shown in Figure 3C. Visual inspection indicated that most of the transitions occurred between States I and II, and State I was associated with the largest fraction of transitions in both AD and HC. However, it is worth noting that AD transitioned from/to State II more frequently than HC did.

### 3.3 Analyses on regional temporal variability

**Figure 4:**
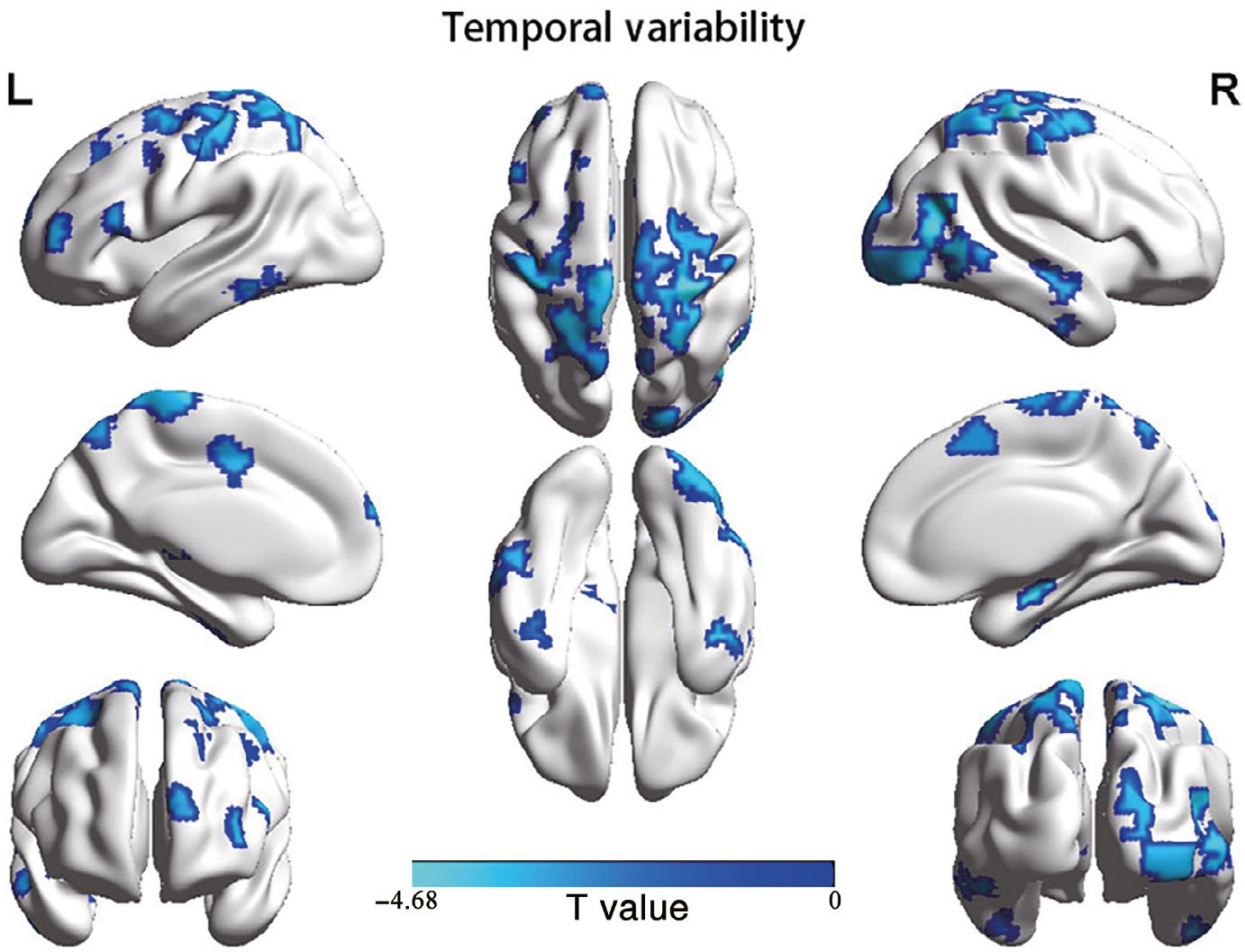
Brain regions showing significant differences in temporal variability between AD and HC (*P* < .05, FDR corrected)

Among the 625 regions in the brain network, 57 regions exhibited significantly decreased temporal variability in AD compared with those of HC (*P* < .05, FDR corrected), which were mainly distributed in the regions of the somatomotor network (SMN), control network (CN), default mode network (DMN), and visual network (VIS) (Figure 4 and Table S1).

### 3.4 Relationship of functional connectivity dynamics with the clinical performance of AD patients

A significant negative correlation was observed between the NPI scores of AD patients and the median strength of the significantly decreased FC (*R* = –0.519, *P* = .023) in State II. A marginally significant positive correlation was observed between the NPI scores and the median strength of the significantly increased FC (*R* = 0.443, *P* = .057) in State II. Moreover, the NPI scores of AD patients also showed a significant negative correlation with regional temporal variability in the left inferior temporal gyrus (*R* = –0.553, *P* = .008), right middle temporal gyrus (*R* = –0.536, *P* = .010), right caudate nucleus (*R* = –0.521, *P* = .013), and left superior temporal gyrus (*R* = –0.430, *P* = .046).

### 3.5 Validation results

We validated the reliability of our main findings with respect to the influence of head motion. After using regression to correct for head motion, three reoccurring dynamic FC states were extracted, and they resembled those found in the main analyses (Figure S1). Similarly, the ROC analyses based on FC with significant between‐group differences achieved good performance (AUC ≥ 0.99). The between‐group comparison of the mean dwell time of the three states also produced highly compatible results (Figure S2). With respect to the regional temporal variability, 55 of the 57 regions that showed significant decreases in the main analyses had recovered in the validation analyses. In addition, the validation analyses found some additional regions with significantly decreased regional temporal variability, mainly in the SMN and frontal cortex (Figure S3 and Table S2).

## 4 DISCUSSION

Our main findings are as follows. First, three reoccurring dynamic FC states were identified. States I and II both contained FC with statistically significant between‐group differences, which may be helpful to identify AD. Moreover, the median strength of FC with significant between‐group differences in State II was significantly correlated with the NPI scores of AD patients. Second, the mean dwell time of AD was significantly shorter in State I but significantly longer in State II than HC. Third, AD had significantly lower temporal variability than HC, mainly in the regions of the SMN, CN, DMN, and VIS. Among them, the temporal variability of the left inferior temporal gyrus, right middle temporal gyrus, right caudate nucleus, and left superior temporal gyrus was significantly correlated with the NPI scores of AD patients.

### 4.1 Dynamic functional connectivity states

The three reoccurring FC states varied in overall connectivity strength and occurrence frequency. To facilitate descriptions and interpretation, we named the three states based on their characteristics. State I was named the baseline state because it had the lowest overall connectivity strength and was the only state that presented in every participant. In contrast, State III was named the spiking state because it had the highest overall connectivity strength and presented in less than one‐third of the participants. Compared with States I and III, State II had moderate overall connectivity strength and presented in nearly all the AD but only half of the HC. In addition, State II had the largest number of connections that showed significant differences between the AD and the HC. According to these results, State II was named the AD‐abnormality state.

Except for the rare spiking state, the baseline state and the AD‐abnormality state both contained FC with significant between‐group differences. The locations of these connections agreed well with the regions where abnormalities have been repeatedly reported in previous neuroimaging studies on AD, including the hippocampus,^32^, ^33^ amygdala,^33^ insula^9^, ^33^ medial temporal cortex,^9^, ^33^ and fontal cortex.^33^, ^34^ Intriguingly, the signs of these FCs were often opposite between the two groups. Combined with the results of the ROC analyses, we speculated that FC with significant between‐group differences might serve as an effective biomarker for AD. Such findings were also consistent with previous studies that suggested FC dynamics might reflect distinct features of neural system functional capacity,^35^, ^36^ and thus offer suggestions for potential biomarkers of neurological diseases overlooked by stationary FC analyses.^11^, ^37^

### 4.2 Temporal properties of dynamic functional connectivity states

The finding that the most loosely connected baseline state had the highest occurrence was also reported by previous studies.^12^, ^38^ Since FC in the resting state was suggested to support information transfer between brain regions,^39^, ^40^ the high occurrence of the baseline state may suggest that the human brain prefers switching to a state where information transfer is less efficient but probably more energy saving.^41^ This finding is also consistent with previous studies that considered aging as a main cause for longer dwell time in weaker FC states.^38^, ^42^ Compared with HC, AD patients spent less time in the baseline state with weak overall connectivity but more time in the relatively stronger AD‐abnormality state. This result partially supports the findings of previous stationary FC analyses that AD had increased FC, probably the result of a compensating response to the degenerated FC.^43^, ^44^ This result also agrees with a recent study on FC dynamics in AD, which used independent component analysis to derive the nodes of the functional brain network and reported shorter dwell time on the weakest state compared to HC.^16^ Notably, a study that used a similar method found the opposite pattern.^18^ This discrepancy could be attributed to the instability of the independent component analysis method, in which it is inherently difficulty to determine the number of independent components and distinguish the true signal from noise.^40^ Additionally, we found that the median strength of the FC with significant between‐group differences in the AD‐abnormality state was significantly correlated with the NPI scores of AD patients. Specifically, the significantly increased FC had a positive correlation with the NPI, while the significantly decreased FC had a negative correlation with the NPI. Our findings were thus supportive of the suggestion that connectivity changes in dynamic FC states may be behaviorally relevant.^45^ In addition, since the NPI score can reflect the severity of neuropsychiatric characteristics in AD, our findings also imply that the AD‐abnormality state may be closely associated with aberrant motor behaviors and neuropsychiatric symptoms, such as apathy, disinhibition, and dysphoria in AD patients. The longer dwell time on the AD‐abnormality state in AD suggested that it may cost them more energy to cope with neuropsychiatric symptoms. Taking the above findings together, we concluded that the AD‐abnormality state might be a specific, core working state of AD.

Additionally, we also observed that the state transition number of AD was larger than that of HC, indicating that AD transitioned more frequently than HC did. Furthermore, the distribution of transition frequencies showed that AD was more likely to transition between the AD‐abnormality state and the baseline state than HC. We speculated that AD may not have enough energy to transition to the spiking state, which could be partially supported by a growing number of studies that have consistently reported hypometabolism in AD.^46^, ^47^, ^48^

### 4.3 Regional temporal variability and its correlations with clinical scores

Although AD experienced more state transitions than HC, the regional temporal variability of AD was lower than that of HC, probably because the FC matrices derived from the AD group tended to have lower overall diversity due to a common disease, despite that they were clustered into different states. The regions with decreased temporal variability were mainly distributed in the SMN, CN, DMN, and VIS, consistent with previous findings.^5^, ^8^, ^49^, ^50^ The decreased temporal variability in the SMN and VIS could be related to reduced flexibility in sensory, motor, and visual functions. For the DMN and CN, the decreased temporary variability might be associated with impaired cognitive functions, as the DMN plays a pivotal role in necessary cognitive processes^51^ and especially influences memory consolidation,^8^ and the CN is involved in cognitive control and has also been reported to have hypoconnectivity during AD progression.^49^ In addition, a significantly negative correlation was found between the NPI scores of the AD patients and the regional temporal variability of the left inferior temporal cortex, right middle temporal cortex, left caudate lobe, and left superior frontal cortex. Thus, we suggest that regional temporal variability might be a potential biomarker to distinguish AD from HC.

### 4.4 Limitations and further considerations

The present work has a few limitations that should be noted. First, although our results suggested that the AD‐abnormality state might be a core and specific state of AD, further confirmatory studies in larger datasets are needed. Second, since previous studies have found different levels of brain damage at different diagnostic stages of AD,^52^ FC dynamics may also exhibit different characteristics as the disease progresses. As the initial goal was to investigate the FC dynamics in AD, the diagnostic stages were not considered in this article. A prominent future direction would be to investigate the change in FC dynamics during the progression of AD. Finally, we adopted the widely used sliding‐window approach to extract FC dynamics in the current study. To avoid potential bias, future studies can consider using other extraction methods, such as the point‐process method,^53^ to analyze FC dynamics in AD.

## CONFLICT OF INTEREST

None of the authors has any conflicts of interest to disclosure.

## Supporting information

Supplementary Material
