# Supplementary material for: Abnormal dynamic functional connectivity in Alzheimer’s disease
Source: CNS Neurosci Ther. 2020 May 6;26(9):962–71. doi: 10.1111/cns.13387 (PMC7415210; doi:10.1111/cns.13387)
Supplement: Supplementary file 1 — Supplementary Material [file CNS-26-962-s001.docx]

**Supplemental Information for**

**“Abnormal Dynamic Functional Connectivity in Alzheimer’s Disease”**

Yue Gu^1#^, Ying Lin^1#^, Liangliang Huang^1^, Junji Ma^1^, Jinbo Zhang^1^, Yu Xiao^1^, Zhengjia Dai^1*^, for the Alzheimer's Disease Neuroimaging Initiative^a^

^1^Department of Psychology, Sun Yat-sen University, Guangzhou, China, 510006

^a^Data used in preparation of this article were obtained from the Alzheimer's Disease Neuroimaging Initiative (ADNI) database (adni.loni.usc.edu). As such, the investigators within the ADNI contributed to the design and implementation of ADNI and/or provided data but did not participate in analysis or writing of this report. A complete listing of ADNI investigators can be found at: http://adni.loni.usc.edu/wp-content/uploads/how_to_apply/ADNI_Acknowledgement_List.pdf.

^#^ Yue Gu and Ying Lin contributed equally to this work.

Running title: Dynamic Functional connectivity in AD

*Corresponding Authors:

Zhengjia Dai, Ph.D., Department of Psychology, Sun Yat-sen University, Guangzhou, China, 510006. Email: daizhengj@mail.sysu.edu.cn

The supplemental information contains the clustering results under different settings of the cluster number, detailed results of regional temporal variability analyses, and all the validation results.

*Detailed results of the regional temporal analyses*

**Table S1** List of the brain regions where AD showed significantly decreased temporal variability compared to HC (*P* < 0.05, FDR corrected).

| Region | *T* | *P* |
| --- | --- | --- |
| Postcentral_R | -4.676 | < ${10}^{-3}$ |
| Postcentral_L | -4.415 | < ${10}^{-3}$ |
| Temporal_Mid_R | -4.292 | < ${10}^{-3}$ |
| Postcentral_R | -4.252 | < ${10}^{-3}$ |
| Hippocampus_R | -4.174 | < ${10}^{-3}$ |
| Paracentral_Lobule_L | -4.151 | < ${10}^{-3}$ |
| Temporal_Inf_R | -4.062 | < ${10}^{-3}$ |
| Temporal_Mid_R | -4.052 | < ${10}^{-3}$ |
| Temporal_Inf_R | -4.026 | < ${10}^{-3}$ |
| Temporal_Mid_R | -4.005 | < ${10}^{-3}$ |
| Parietal_Sup_R | -3.896 | < ${10}^{-3}$ |
| Precuneus_R | -3.836 | < ${10}^{-3}$ |
| Parietal_Sup_R | -3.785 | < ${10}^{-3}$ |
| Parietal_Sup_L | -3.758 | < ${10}^{-3}$ |
| Parietal_Sup_L | -3.754 | < ${10}^{-3}$ |
| Precentral_R | -3.739 | < ${10}^{-3}$ |
| Occipital_Inf_R | -3.726 | < ${10}^{-3}$ |
| Frontal_Sup_L | -3.644 | < ${10}^{-3}$ |
| Frontal_Sup_R | -3.591 | < ${10}^{-3}$ |
| Postcentral_L | -3.563 | < ${10}^{-3}$ |
| Occipital_Inf_R | -3.560 | < ${10}^{-3}$ |
| Precuneus_L | -3.554 | < ${10}^{-3}$ |
| Paracentral_Lobule_L | -3.512 | < ${10}^{-3}$ |
| Temporal_Inf_L | -3.508 | < ${10}^{-3}$ |
| Postcentral_R | -3.474 | 0.001 |
| Occipital_Inf_R | -3.471 | 0.001 |
| Frontal_Mid_L | -3.461 | 0.001 |
| Thalamus_L | -3.417 | 0.001 |
| Paracentral_Lobule_R | -3.397 | 0.001 |
| Parietal_Sup_L | -3.368 | 0.001 |
| Precuneus_L | -3.358 | 0.002 |
| Precentral_L | -3.349 | 0.002 |
| Occipital_Sup_R | -3.347 | 0.002 |
| Precentral_R | -3.335 | 0.002 |
| Temporal_Inf_L | -3.311 | 0.002 |
| Cingulum_Mid_L | -3.284 | 0.002 |
| Precuneus_L | -3.268 | 0.002 |
| Caudate_L | -3.228 | 0.002 |
| Frontal_Inf_Tri_L | -3.198 | 0.002 |
| Postcentral_R | -3.194 | 0.002 |
| Precentral_L | -3.181 | 0.003 |
| Frontal_Sup_Medial_L | -3.178 | 0.003 |
| Temporal_Inf_L | -3.161 | 0.003 |
| Precentral_R | -3.127 | 0.003 |
| Temporal_Mid_R | -3.120 | 0.003 |
| Parietal_Sup_L | -3.107 | 0.003 |
| Postcentral_L | -3.101 | 0.003 |
| Supp_Motor_Area_R | -3.074 | 0.003 |
| Temporal_Mid_R | -3.071 | 0.004 |
| Temporal_Inf_L | -3.050 | 0.004 |
| Precentral_R | -3.045 | 0.004 |
| Frontal_Sup_L | -3.025 | 0.004 |
| Supp_Motor_Area_R | -3.019 | 0.004 |
| Precuneus_L | -3.018 | 0.004 |
| Frontal_Sup_L | -3.012 | 0.004 |
| Precuneus_R | -3.004 | 0.004 |
| Parietal_Sup_R | -2.993 | 0.004 |

*Validation results*

*
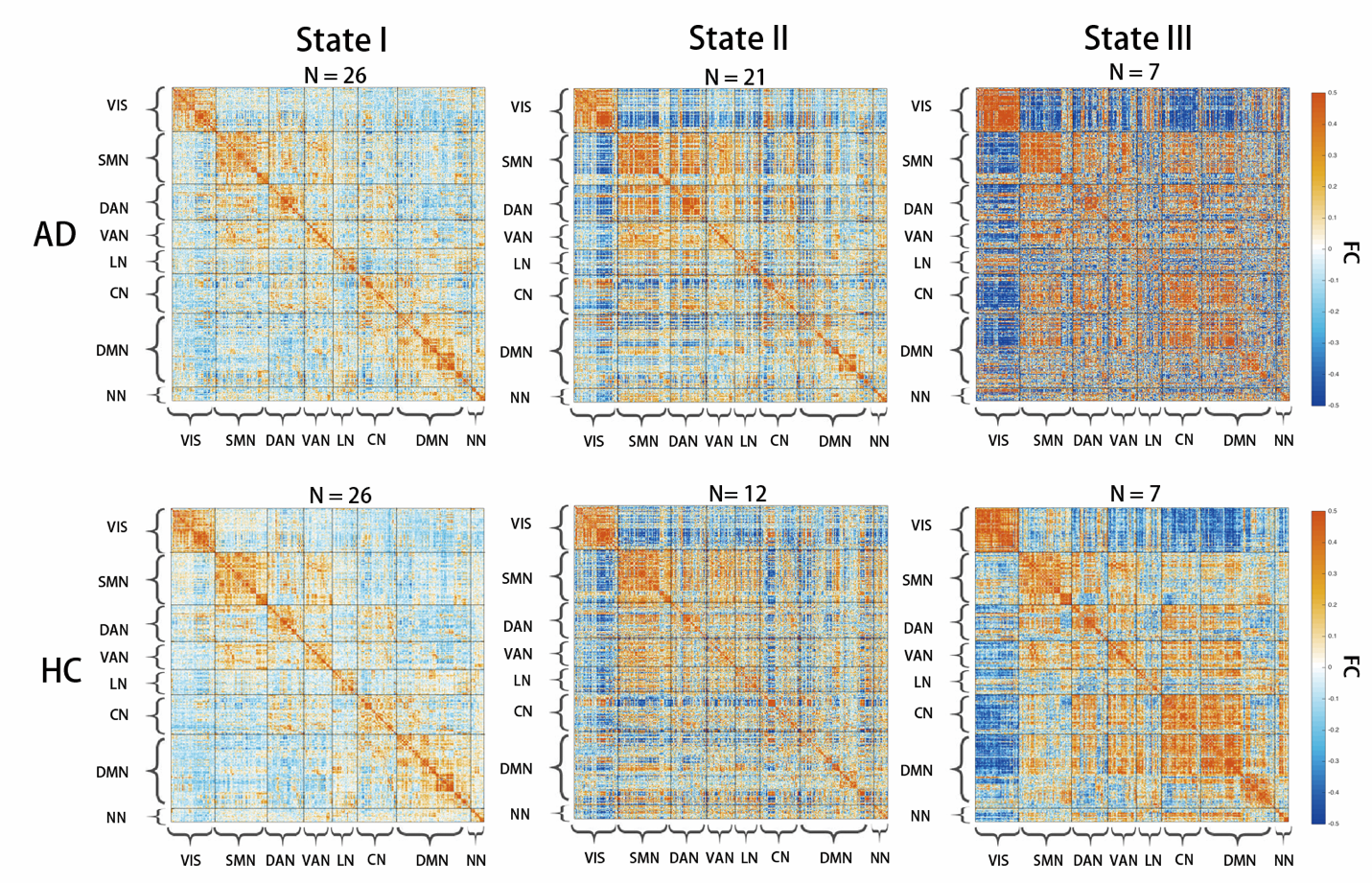
*

**Figure S1** Validation results: Median FC matrices of the three state in the AD and HC groups. N is the number of participants that the corresponding state occurred in. Each matrix is organized according to the functional modules extracted by Yeo et al.,^54^ which divides the 625 regions into seven networks and one uncertain part. VIS = visual; SMN = somatomotor; DAN = dorsal attention; VAN = ventral attention; LN = limbic; CN = control; DMN = default network; NN = uncertain part.


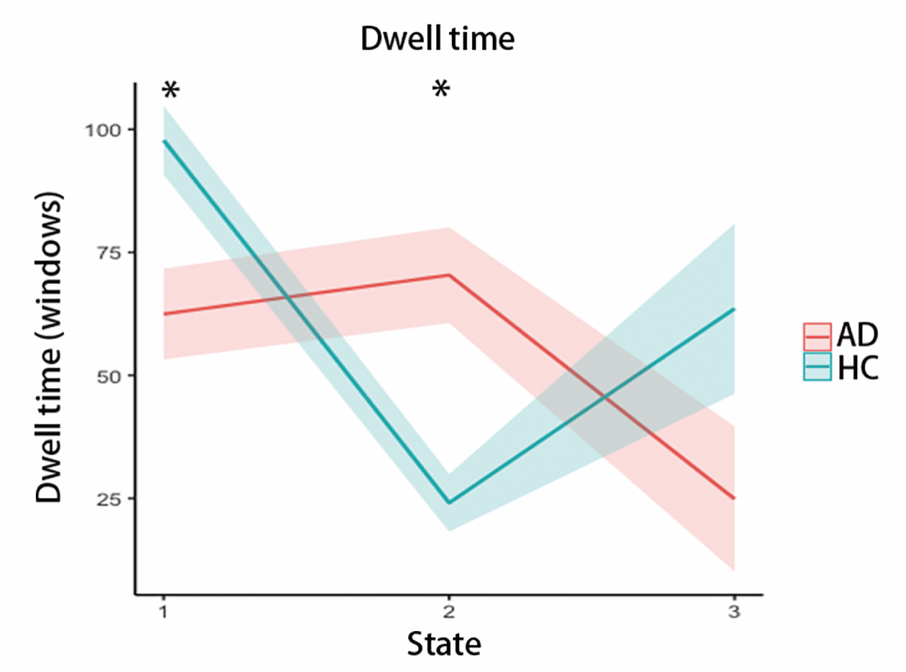


**Figure S2** Validation results: Comparison of the mean dwell time of each state, where the shadows indicate the standard error of mean (SEM) over the corresponding group and the asterisks indicate significant between-group difference (P < 0.05, FDR corrected).


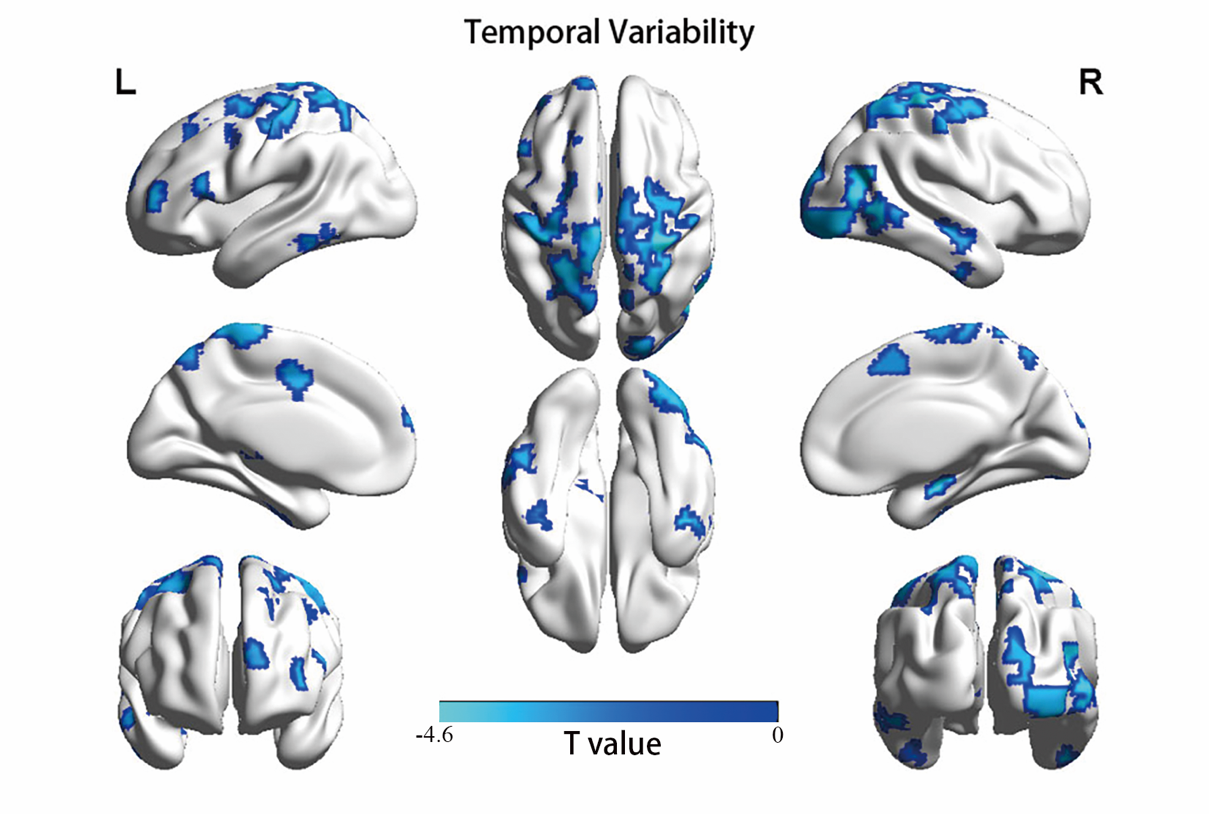


**Figure S3** Validation results: Brain regions showing significant differences in temporal variability between AD patients and HCs (*P* < 0.05, FDR corrected). The lighter blue in figure means larger *T* values, which indicates more significant decrement.

**Table S2** Validation results: List of the brain regions where AD showed significantly decreased temporal variability compared to HC (*P* < 0.05, FDR corrected). The regions that were also found in the main analyses are shown in **bold**, while the regions that were found in the main analyses but missed in the validation analyses are shown in *italic*.

| **Region** | ***T*** | ***P*** |
| --- | --- | --- |
| **Parietal_Sup_R** | **-4.599** | **<** $\boldsymbol{10}^{\boldsymbol{-3}}$ |
| **Temporal_Inf_R** | **-4.543** | **<** $\boldsymbol{10}^{\boldsymbol{-3}}$ |
| **Postcentral_R** | **-4.448** | **<** $\boldsymbol{10}^{\boldsymbol{-3}}$ |
| **Temporal_Mid_R** | **-4.311** | **<** $\boldsymbol{10}^{\boldsymbol{-3}}$ |
| **Postcentral_R** | **-4.164** | **<** $\boldsymbol{10}^{\boldsymbol{-3}}$ |
| **Parietal_Sup_L** | **-4.158** | **<** $\boldsymbol{10}^{\boldsymbol{-3}}$ |
| **Temporal_Mid_R** | **-4.130** | **<** $\boldsymbol{10}^{\boldsymbol{-3}}$ |
| **Temporal_Inf_R** | **-4.094** | **<** $\boldsymbol{10}^{\boldsymbol{-3}}$ |
| **Precentral_R** | **-4.053** | **<** $\boldsymbol{10}^{\boldsymbol{-3}}$ |
| **Temporal_Mid_R** | **-4.045** | **<** $\boldsymbol{10}^{\boldsymbol{-3}}$ |
| **Postcentral_L** | **-4.043** | **<** $\boldsymbol{10}^{\boldsymbol{-3}}$ |
| **Postcentral_L** | **-3.796** | **<** $\boldsymbol{10}^{\boldsymbol{-3}}$ |
| **Occipital_Inf_R** | **-3.785** | **<** $\boldsymbol{10}^{\boldsymbol{-3}}$ |
| **Precentral_R** | -3.775 | < ${10}^{-3}$ |
| **Hippocampus_R** | **-3.753** | **<** $\boldsymbol{10}^{\boldsymbol{-3}}$ |
| **Parietal_Sup_R** | **-3.720** | **0.001** |
| **Paracentral_Lobule_L** | **-3.707** | **0.001** |
| **Occipital_Inf_R** | **-3.702** | **0.001** |
| **Parietal_Sup_L** | **-3.674** | **0.001** |
| Paracentral_Lobule_L | -3.592 | 0.001 |
| **Precentral_L** | **-3.587** | **0.001** |
| **Frontal_Sup_Medial_L** | **-3.580** | **0.001** |
| **Precuneus_L** | **-3.566** | **0.001** |
| **Occipital_Inf_R** | **-3.560** | **0.001** |
| **Postcentral_R** | **-3.555** | **0.001** |
| **Frontal_Sup_R** | **-3.539** | **0.001** |
| **Parietal_Sup_R** | **-3.516** | **0.001** |
| **Frontal_Sup_L** | **-3.514** | **0.001** |
| **Temporal_Mid_R** | **-3.487** | **0.001** |
| **Precentral_R** | **-3.465** | **0.001** |
| Frontal_Mid_L | -3.452 | 0.001 |
| **Temporal_Mid_R** | **-3.441** | **0.001** |
| Frontal_Mid_L | -3.439 | 0.001 |
| Frontal_Sup_R | -3.439 | 0.001 |
| **Precuneus_R** | **-3.437** | **0.001** |
| **Frontal_Sup_L** | **-3.429** | **0.001** |
| Temporal_Mid_R | -3.427 | 0.001 |
| **Temporal_Inf_L** | **-3.389** | **0.001** |
| **Parietal_Sup_L** | **-3.367** | **0.002** |
| Precentral_R | -3.364 | 0.002 |
| Occipital_Mid_R | -3.341 | 0.002 |
| Precentral_R | -3.337 | 0.002 |
| Lingual_R | -3.334 | 0.002 |
| Frontal_Mid_L | -3.321 | 0.002 |
| **Postcentral_R** | **-3.309** | **0.002** |
| **Precuneus_L** | **-3.290** | **0.002** |
| **Precuneus_L** | **-3.288** | **0.002** |
| Frontal_Sup_L | -3.269 | 0.002 |
| **Temporal_Inf_L** | **-3.266** | **0.002** |
| **Precentral_R** | **-3.262** | **0.002** |
| **Paracentral_Lobule_R** | **-3.258** | **0.002** |
| Precentral_L | -3.254 | 0.002 |
| **Precentral_L** | **-3.227** | **0.002** |
| Putamen_L | -3.213 | 0.002 |
| Frontal_Mid_L | -3.210 | 0.002 |
| Frontal_Sup_R | -3.208 | 0.002 |
| Occipital_Mid_R | -3.160 | 0.003 |
| **Parietal_Sup_L** | **-3.149** | **0.003** |
| **Frontal_Sup_L** | **-3.141** | **0.003** |
| Precentral_L | -3.136 | 0.003 |
| **Postcentral_L** | **-3.125** | **0.003** |
| Fusiform_R | -3.105 | 0.003 |
| **Caudate_L** | **-3.085** | **0.003** |
| Calcarine_L | -3.077 | 0.003 |
| Precentral_L | -3.076 | 0.003 |
| **Temporal_Inf_L** | **-3.074** | **0.003** |
| **Precentral_R** | **-3.067** | **0.004** |
| Pallidum_R | -3.065 | 0.004 |
| Supp_Motor_Area_L | -3.055 | 0.004 |
| Supp_Motor_Area_L | -3.055 | 0.004 |
| Occipital_Mid_R | -3.050 | 0.004 |
| Parietal_Sup_R | -3.045 | 0.004 |
| Occipital_Inf_L | -3.043 | 0.004 |
| Occipital_Mid_L | -3.039 | 0.004 |
| Occipital_Mid_R | -3.037 | 0.004 |
| Frontal_Mid_R | -3.031 | 0.004 |
| Fusiform_R | -3.019 | 0.004 |
| Temporal_Inf_R | -3.015 | 0.004 |
| **Supp_Motor_Area_R** | **-2.999** | **0.004** |
| Frontal_Mid_L | -2.991 | 0.004 |
| Precentral_R | -2.983 | 0.004 |
| **Thalamus_L** | **-2.973** | **0.005** |
| Temporal_Inf_L | -2.968 | 0.005 |
| **Temporal_Inf_L** | **-2.961** | **0.005** |
| Rectus_L | -2.959 | 0.005 |
| **Occipital_Sup_R** | **-2.939** | **0.005** |
| Temporal_Inf_L | -2.938 | 0.005 |
| Precentral_L | -2.933 | 0.005 |
| Frontal_Sup_Medial_R | -2.932 | 0.005 |
| SupraMarginal_R | -2.931 | 0.005 |
| Parietal_Inf_L | -2.930 | 0.005 |
| Temporal_Inf_R | -2.927 | 0.005 |
| Parietal_Sup_R | -2.910 | 0.005 |
| Cingulum_Mid_L | -2.901 | 0.006 |
| **Cingulum_Mid_L** | **-2.900** | **0.006** |
| Frontal_Mid_R | -2.897 | 0.006 |
| **Precuneus_L** | **-2.878** | **0.006** |
| Temporal_Inf_R | -2.870 | 0.006 |
| Frontal_Mid_L | -2.869 | 0.006 |
| Caudate_L | -2.869 | 0.006 |
| Hippocampus_R | -2.862 | 0.006 |
| **Frontal_Mid_L** | **-2.841** | **0.007** |
| Precentral_L | -2.839 | 0.007 |
| Frontal_Sup_L | -2.834 | 0.007 |
| Temporal_Mid_R | -2.816 | 0.007 |
| Occipital_Mid_L | -2.810 | 0.007 |
| Hippocampus_L | -2.800 | 0.007 |
| Occipital_Mid_L | -2.800 | 0.007 |
| Frontal_Mid_Orb_L | -2.799 | 0.007 |
| **Paracentral_Lobule_L** | **-2.793** | **0.007** |
| Calcarine_L | -2.788 | 0.008 |
| Frontal_Inf_Tri_R | -2.786 | 0.008 |
| Frontal_Mid_Orb_L | -2.778 | 0.008 |
| Supp_Motor_Area_R | -2.777 | 0.008 |
| Fusiform_L | -2.777 | 0.008 |
| Occipital_Mid_L | -2.776 | 0.008 |
| Frontal_Inf_Tri_L | -2.768 | 0.008 |
| Frontal_Mid_L | -2.768 | 0.008 |
| Precuneus_L | -2.768 | 0.008 |
| ParaHippocampal_R | -2.762 | 0.008 |
| Frontal_Sup_R | -2.737 | 0.009 |
| **Precuneus_R** | **-2.725** | **0.009** |
| Fusiform_L | -2.724 | 0.009 |
| Precentral_L | -2.718 | 0.009 |
| Rectus_L | -2.716 | 0.009 |
| Postcentral_R | -2.712 | 0.009 |
| Precentral_R | -2.701 | 0.010 |
| Frontal_Mid_R | -2.701 | 0.010 |
| Occipital_Sup_L | -2.699 | 0.010 |
| Postcentral_R | -2.696 | 0.010 |
| Frontal_Mid_R | -2.680 | 0.010 |
| *Frontal_Inf_Tri_L* | *-2.668* | *0.010* |
| *Supp_Motor_Area_R* | *-2.449* | *0.018* |
